# Dissipative Kerr single soliton generation with extremely high probability via spectral mode depletion

**DOI:** 10.1007/s12200-022-00047-y

**Published:** 2022-12-01

**Authors:** Boqing Zhang, Nuo Chen, Xinda Lu, Yuntian Chen, Xinliang Zhang, Jing Xu

**Affiliations:** 1 School of Optical and Electronic Information, Huazhong University of Science and Technology, Wuhan, 430074 China; 2 Wuhan National Laboratory for Optoelectronics, Huazhong University of Science and Technology, Wuhan, 430074 China

**Keywords:** Kerr soliton, Single soliton generation, Spectral filtering

## Abstract

Optical Kerr solitons generation based on microresonators is essential in nonlinear optics. Among various soliton generation processes, the single soliton generation plays a pivotal role since it ensures rigorous mode-locking on each comb line whose interval equals the free spectral range (FSR) of the microresonator. Current studies show that single soliton generation is challenging due to cavity instability. Here, we propose a new method to greatly improve single soliton generation probalility in the anomalous group velocity dispersion (GVD) regime in a micro-ring resonator based on silicon nitride. The improvement is realized by introducing mode depletion through an integrated coupled filter. It is convenient to introduce controllable single mode depletion in a micro-ring resonator by adjusting the response function of a coupled filter. We show that spectral mode depletion (SMD) can significantly boost the single soliton generation probability. The effect of SMD on the dynamics of optical Kerr solitons generation are also discussed. The proposed method offers a straightforward and simple way to facilitate robust single soliton generation, and will have an impact on the research development in optical Kerr soliton generation and on-chip optical frequency mode manipulation.

**Graphical abstract:**

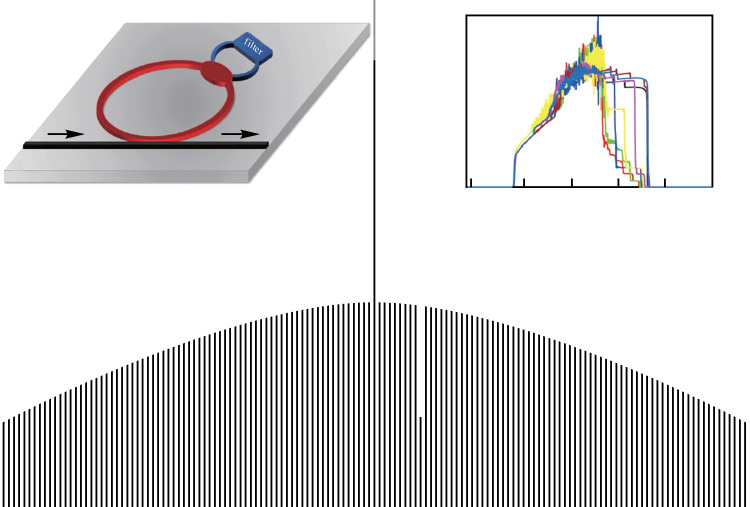

## Introduction

Optical frequency comb (OFC), which enables frequency measurement with high precision in fundamental and applied sciences, has been extensively explored theoretically and experimentally on many platforms. Particularly, on-chip dissipative Kerr soliton (DKS) is promising to achieve highly compact, chip-scale broadband comb sources at microwave-to-terahertz repetition rates with low power consumption [1, 2]. Microresonator-based DKS has shown broad prospects for various applications, including spectroscopy [3–5], precise time measurement (i.e., optical clock) [6], soliton communications [7], and fast-detecting LIDAR [8, 9]. In various DKS generation processes, bright single soliton generation in the anomalous group-velocity dispersion (GVD) regime is up-and-coming for many emerging applications. It ensures rigorous mode-locking between each resonance with an interval equal to the free spectral range (FSR) of the microresonator [10]. Specifically, a single soliton propagating in a microresonator can be characterized by an impeccably smooth *Sech*^*2*^ spectrum, while a multi-soliton exhibits a complex temporal waveform and an unsmooth spectrum due to the large intensity variations between the comb lines. In general, the number of solitons in a microcavity is random. Therefore, finding an effective method for on-chip deterministic single soliton generation is necessary.

Deterministic single soliton generation is very challenging. However, several efforts toward deterministic single soliton generation have paved the way to solve the existing problems. For instance, deterministic single cavity soliton generation can be obtained by backward tuning the pump frequency [11]. Nonetheless, this method requires accurate tuning speed. In another work, researchers showed that spatial mode interaction could be used to shift resonance frequency and change the local dispersion, resulting in deterministic single soliton generation [12]. However, improper local dispersion engineering can be detrimental to the stability of solitons [13]. Furthermore, mode interaction-induced GVD discontinuity can be utilized to suppress or enhance individual comb lines [14]. This process is akin to using the inverse-Kelly sideband to introduce background fluctuation, thereby driving the Kerr combs to the single soliton state [15]. However, the operation conditions are very critical. E.g., large detuning is required, and it only works in the small dispersion region. An intriguing method of fundamental-second-harmonic mode coupling for soliton regulation was proposed and used for deterministic single soliton transition [16]. Nevertheless, it is well-known that ${\chi }^{(2)}$ nonlinearity is absent in materials with central symmetry. Another attempt using the balance between phase mismatch and third-harmonic-generation coupling strength has been proposed to realize deterministic soliton generation [17]. It is a passive mechanism without introducing additional power consumption. The concerning issue is that the ${\chi }^{(3)}$ nonlinearity is usually very weak.

On the other hand, in nonlinear optics, it has been demonstrated that dissipation can counter-intuitively cause modulation instabilities (MIs) in physical systems. Dissipation-induced MIs, also known as the *Gain-through-losses* (GTL) mechanism, have been well addressed in fibers. However, it is elusive in microresonators [18–20]. Moreover, the GTL mechanism is usually utilized to drive the OFC evolving into MIs in the normal GVD regime.

In this paper, we investigate DKS generation based on a micro-ring resonator in the anomalous GVD regime and provide new insight into different dynamic behaviors induced by spectral mode depletion (SMD). Strikingly, single soliton generation with an extremely high probability is observed by simply coupling a filter to the micro-ring resonator, which results in dissipation of a resonant mode. According to the Fourier transform relation, such a depression in the spectrum enhances the fluctuation in the time domain, thus producing a perturbation in the initial state of Kerr combs generation and pushing it to evolve into the single soliton state. Similar phenomena were also observed in recent works concerning such spectrally dependent losses, particularly in the normal dispersion regime [19]. Notably, SMD can be controlled simply by changing the response function of a coupled filter. Numerical simulations based on the generalized Lugiato–Lefever equation (GLLE) [18] show that the probability of single soliton generation is significantly increased, even close to 1. The outstanding performance maintains even under the influence of the thermal effect, showing great robustness of SMD scheme. Additionally, the impact of detuning between the depleted mode and the pump on the single soliton generation and the changes in the soliton existence regime are discussed.

## Principal of operation

### Device structure

**Fig. 1:**
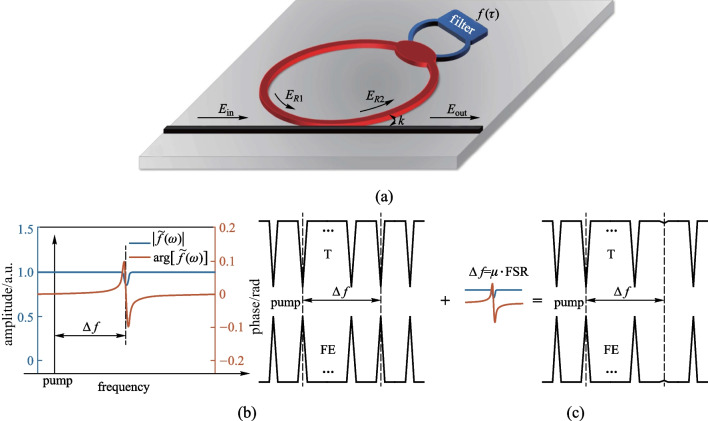
Schematic diagram of the proposed selective SMD system. **a** Device structure. A filter (blue) is coupled to a micro-ring resonator (red). ${E}_{\mathrm{in}}$ and ${E}_{\mathrm{out}}$ are the input and the output electric fields. ${E}_{R1}$ and ${E}_{R2}$ are the electric fields at different position in the resonator, where ${E}_{R1}=a{\mathrm{e}}^{\mathrm{i}\phi }{E}_{R2}$. **b** Amplitude response $\left|\widetilde{f}\left(\omega \right)\right|$ (solid blue line) and phase response $\mathrm{arg}[\widetilde{f}\left(\omega \right)]$ (solid red line) of $\widetilde{f}\left(\omega \right)$, where $\Delta f$ is usually a multiple of FSR. **c** Schematic diagram of the change of *T* and FE before and after introducing the selective SMD

To achieve selective SMD in a micro-ring resonator that has discrete spectral modes with an equal interval, a coupled system consisting of a micro-ring resonator and a lumped filter is proposed, as sketched in Fig. 1a. The micro-ring resonator is also coupled to a bus waveguide, from which the pump light is launched into the resonator. A similar design with optical fibers has been studied, which enables tunable frequency comb generation in the normal GVD region [20]. However, due to the large discrepancy in the cavity length and the spectral mode separation between a fiber ring and a micro-ring, the spectral properties of the two kinds of cavities are fundamentally dissimilar. As a result, the consequences of introducing spectral filtering in a micro-ring resonator remain unclear. In this section, we elaborate on the feasibility of introducing the filtering effect in a micro-ring resonator.

As shown in Fig. 1a, the micro-ring resonator is coupled with a narrow band filter (i.e., the filter bandwidth only covers one resonant peak) with frequency response function $\widetilde{f}\left(\omega \right)$. Considering that the filter is used to introduce considerable depletion of a specific resonance mode in our selective SMD scheme, the spectral reflectivity profile of the filter can be expressed as: $\widetilde{f}\left(\omega \right)=\mathrm{exp}\left[\alpha \left(\omega \right)+\mathrm{i}\psi \left(\omega \right)\right]$, where $\left|\widetilde{f}\left(\omega \right)\right|=1-g\mathrm{exp}\left(-{\left(\omega -{\omega }_{f}\right)}^{2}/{\sigma }_{f}^{2}\right)$ and $\psi \left(\omega \right)=\mathrm{arg}\left[\widetilde{f}\left(\omega \right)\right]=-\mathcal{H}\left[\alpha \left(\omega \right)\right]$ [21], where $\mathcal{H}\left[\alpha \left(\omega \right)\right]$ means the Hilbert transform of $\alpha \left(\omega \right)$ [22]. $g$, ${\omega }_{f}$ and ${\sigma }_{f}$ are the filter strength, the central frequency and the bandwidth of the filter, respectively. In Fig. 1b, the amplitude responses of the filter $\left|\widetilde{f}\left(\omega \right)\right|$ are represented by a Gaussian function, where ${\sigma }_{f}$ equals a quarter of the resonator’s FSR in our simulation (Note that ${\sigma }_{f}$ should be larger than the bandwidth of the resonance while smaller than twice the FSR). ${\omega }_{f}=2\uppi \Delta f$, where $\Delta f=\mu \cdot \mathrm{FSR}$ ($\mu$ is the relative mode number with respect to the pump frequency) is the relative central frequency of the filter. For the proposed structure, the transmittance from the input port to the output port of the micro-ring can be expressed as:

1 $$T=\frac{{E}_{\mathrm{out}}}{{E}_{\mathrm{in}}}=\frac{r - a{\mathrm{e}}^{\mathrm{i}\phi }\widetilde{f}\left(\omega \right)}{1 - ar{\mathrm{e}}^{\mathrm{i}\phi }\widetilde{f}\left(\omega \right) }.$$

Herein, ${E}_{\mathrm{in}}$ and ${E}_{\mathrm{out}}$ are the electric fields at the input and output port, respectively. $a=\mathrm{exp}\left(-{\alpha}_{0}L/2\right)$, where ${\alpha }_{0}$ is the propagation loss coefficient of the micro-ring resonator, and $L$ is the circumference. $\phi =\omega {t}_{\mathrm{R}}$ is the round-trip phase shift, where ${t}_{\mathrm{R}}$ is the round-trip time. Meanwhile, the electric field in a micro-ring resonator can be represented by the field enhancement factor (FE), which is defined as the ratio of the intracavity field to the input field:

2 $$\text{FE}=\frac{{E}_{R2}}{{E}_{\mathrm{in}}}=\frac{\mathrm{i}\kappa a{\mathrm{e}}^{\mathrm{i}\phi }\widetilde{f}\left(\omega \right)}{1 - ar{\mathrm{e}}^{\mathrm{i}\phi }\widetilde{f}\left(\omega \right)}.$$

The square of FE is proportional to the intracavity photon density. It can be seen from Fig. 1c that when the relative central frequency of the filter $\Delta f$ is a multiple of the FSR, the central frequency of the filter will be aligned with a resonant peak. Consequently, the transmittance increases while the FE factor decreases significantly for this resonant mode, which means that this selected mode has been considerably depleted within the resonator, or in other words, selective SMD has been successfully introduced at a specific resonator mode.

### Numerical simulation methods

In this subsection, we introduce the simulation methods of the OFCs generation with SMD. The dynamics of OFC in a micro-ring resonator with a coupled spectral filter can be captured by the GLLE [18]

3 $${t}_{\mathrm{R}}\frac{\partial E\left(t,\tau \right)}{\partial t}=\left[-\alpha -\mathrm{i}\delta -\mathrm{i}L\sum_{k\ge 2}\frac{{\beta }_{k}}{k!}{\left(\mathrm{i}\frac{\partial }{\partial \tau }\right)}^{k}+\mathrm{i}{\gamma L\left|E\right|}^{2}\right]E-f\left(\tau \right)*E+{\sqrt{{\kappa }_{\mathrm{in}}}E}_{\mathrm{in}},$$

where ${t}_{\mathrm{R}}$ is the round-trip time, $E(t,\tau )$ is the intracavity electric field, $\tau$ is the fast time and $t$ is the slow time, which is related to the round-trip index *m* by $E\left(t=m{t}_{\mathrm{R}},\tau \right)=E(0,\tau )$. $\delta$ is the phase detuning between the external pump field ${E}_{\mathrm{in}}$ and the resonant mode of the cavity. To search for soliton states, a tunable laser with center frequency scanning over a resonance of the resonator in decreasing manner is used as pump, so that the pump-resonance detuning $\delta$ is thereby tuned. In the following simulations, the pump laser is linearly tuned with a specific frequency scan speed. Coefficients $\alpha ,{\beta }_{k}, \gamma$ are the intrinsic cavity loss, the *k*th order dispersion coefficients and the Kerr nonlinear coefficient, respectively. ${\kappa }_{\mathrm{in}}$ is the coupling strength between the bus-waveguide and the ring [23]. $f(\tau )$ is the impulse response of the filter, which can be obtained by Inverse Fourier Transform of $\widetilde{f}\left(\omega \right)$. $f(\tau )*E$ is the convolution of $E$ and $f(\tau )$, which describes the depletion effect in the time domain. A standard split-step Fourier method is employed for the numerical simulations using Eq. (3). It is worth noting that the system works in the anomalous GVD regime, and all the simulation parameters are based on the silicon nitride material platform.

## Results and discussion

### Kerr frequency combs and solitons generation

**Fig. 2:**
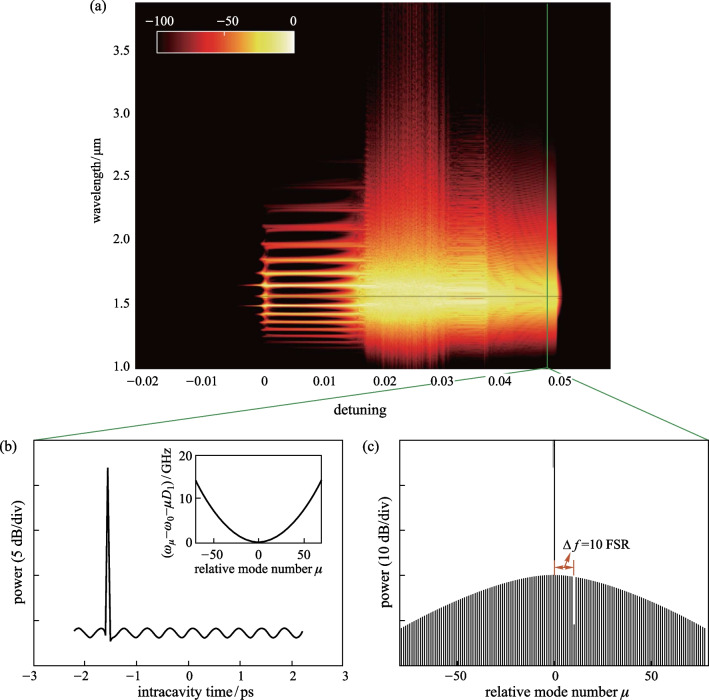
Simulation results of the optical frequency combs with SMD in the micro-ring resonator. **a** Evolution of the comb spectrum with the change of detuning. The comb was initially generated with a pump wavelength of 1550 nm and $\delta$ being tuned from − 0.022 to 0.058. **b** and **c** Illustrate the simulated temporal soliton and spectrum when $\delta$ is tuned to 0.0491. Simulation parameters are ${t}_{\mathrm{R}}=4.4\;\mathrm{ ps}$, $\mathrm{FSR}=226\;\mathrm{ GHz}$, $L=628\;\upmu\mathrm{m}$, $\alpha =9\times 1{0}^{-3}$, ${\kappa }_{\mathrm{in}}=9\times 1{0}^{-3}$, ${\beta }_{2}=-20\;\mathrm{ps}^{2}/\mathrm{km}$, $\gamma =1\; {\left(\mathrm{W}\cdot \mathrm{m}\right)}^{-1}$, ${\left|{E}_{\mathrm{in}}\right|}^{2}=0.58\;\mathrm{ W}$ and $\delta$ scans linearly from − 0.022 at a scan rate of ${10}^{-6}$ per round-trip. The parameters of the coupled filter are $g=0.15$, $\mu =10$, ${\sigma }_{f}=\frac{1}{4}\mathrm{FSR}$. The inset in (**b**) is the dispersion curve of the local cavity modes

Figure 2a shows the simulation results of the comb spectra with pump-resonance detuning $\delta$ tuned from − 0.022 to 0.058 linearly. It can be observed that a low-noise state can be achieved for all the combs and the single soliton can be generated when $\delta$ is tuned to 0.0491. The intracavity waveform and corresponding comb spectrum are illustrated in Fig. 2b, c, respectively. A depression in the smooth *Sech*^*2*^ spectrum can be observed at a specific mode that is aligned with the central frequency of the coupled filter. Meanwhile, the intracavity waveform will also have an oscillating tail due to the beat between the pump and the depleted mode. Similar to the nonlinear loss mechanism introduced by Cherenkov radiation [12], the oscillating tail enables temporal waveforms to fall on the envelopes of solitons, thus facilitating the generation of single soliton states [24].

**Fig. 3:**
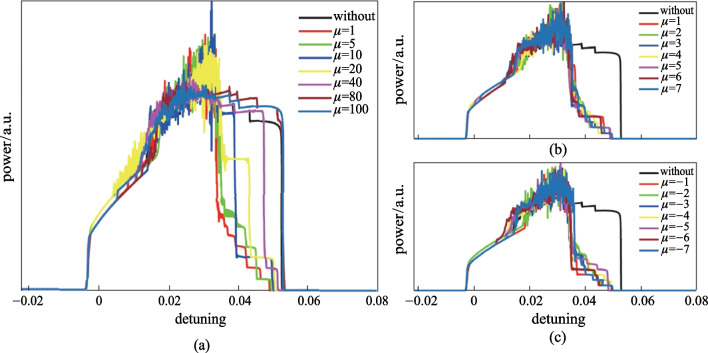
**a** Intracavity power traces with respect to δ for different $\mu$. The pump-resonance detuning $\delta$ ranges from − 0.022 to 0.08. The black solid line is the curve for a micro-ring resonator without the coupled filter. **b** and **c** Are the intracavity power as a function of $\delta$ for positive and negative value of $\mu$, which means central frequency of the filter is located at the blue side or red side of the pump frequency. Simulation parameters are the same as in Fig. 2

Figure 3a illustrates the intracavity power as a function of pump-resonance detuning $\delta$ for different values of $\mu$, which represents the spectral separation of the depleted mode and the pump. Single soliton power is the lowest step in the soliton regime. Soliton regime is the step-shaped power area. It is clear that when $\mu$ is small, e.g., $\mu =1, 5, 10$, that is, the depleted mode is close to the pump, mode depletion introduced by the coupled filter can facilitate the generation of a single soliton state easily. However, when $\mu$ is large, e.g., $\mu =20, 40$, that is, the depleted mode is far from the pump, the generation of a single soliton state gradually diminishes because the energy of mode $\mu$ is small and the influence of single mode depletion is weak. When $\mu$ is very large, e.g., $\mu = 80, 100$, the effect of single mode depletion can even be ignored. The intracavity power is then analogous to that of a micro-ring resonator without the coupled filter. Figure 3b, c show the intracavity powers as a function of $\delta$ for positive and negative value of $\mu$, respectively. It is shown that the generation of single soliton state is regardless of the red or blue detuning of the depleted mode relative to the pump, as there is no physical difference between the two cases in essence.

### Improved single soliton generation

**Fig. 4:**
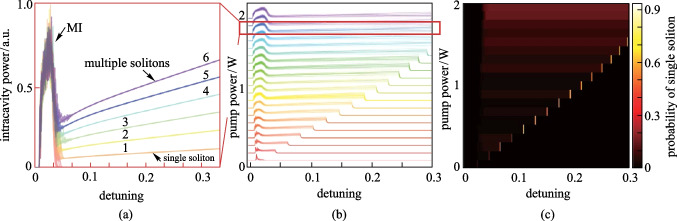
a Simulations results of intracavity power traces under 1.8 W pump power. SMD is not applied and 100 simulation results are presented. Different intracavity soliton numbers are expressed by different colors. **b** Superpositions of intracavity power traces. The pump power is changed from 0.1 to 2 W with a step of 0.1 W to capture the soliton existence region. **a** is an example extracted from (**b**) at 1.8 W pump power, as indicated by the red square. **c** Probability of the single soliton generation vs. pump-resonance detuning and pump power. The parameters used are ${t}_{\mathrm{R}}=4.4\;\mathrm{ ps}$, $L=628\;\upmu\mathrm{m}$, $\alpha =2.4\times 1{0}^{-3}$, ${\beta }_{2}=-81\;\mathrm{ps}^{2}/\mathrm{km}$, $\gamma =1\; {\left(\mathrm{W}\cdot \mathrm{m}\right)}^{-1}$, $\sqrt{{\kappa }_{\mathrm{in}}}=0.0194$. $\delta$ scans linearly starting from − 0.0041 at a speed of $7.55\times {10}^{-7}$ per round-trip

In this section, we discuss single soliton generation probability with selective SMD effect. Figure 4a draws 100 simulations of intracavity power evolution without SMD. With the pump laser scanning across a resonance, the evolution of intracavity power exhibits various physical processes with the accumulation of light field in the ring, from MIs to the soliton regime [25]. Figure 4a reveals that a stable soliton regime appears after the MIs, where the curves merge into several distinct traces representing specific soliton states. Different soliton states are represented by different colors in Fig. 4a. Figure 4a is an example obtained with an injected pump power of 1.8 W. In Fig. 4b, different sets of intracavity power traces are obtained by scanning the pump power from 0 to 2 W, with a step of 0.1 W. Finally, the probability of single soliton generation for each combination of pump power and $\delta$ can be obtained by counting the single soliton generation events among the 100 simulation results. The single soliton generation probabilities for different combinations of pump power and $\delta$ are mapped as in Fig. 4c. It is clearly shown from Fig. 4c that, without mode depletion, the single soliton generation is generally a small probability event, especially for low pump powers.

Next, the SMD enhanced single soliton generation is discussed. Mode depletion in the frequency domain is a consequence of resonant condition change, i.e., the depleted mode is no longer resonant at the specific frequency [26]. In Section 2, we have already demonstrated the feasibility of selective SMD. For a comb-like spectrum, depletion of a resonant mode can introduce a corresponding perturbation to the temporal waveform, resulting in timing jitter of the pulse according to the Fourier transform relation. The perturbation is relatively weak since the energy dissipated at one resonant mode is just a tiny part of the total intracavity energy. Nevertheless, this minor perturbation has a profound impact on the soliton generation, driving the Kerr combs to evolve faster and enter the single soliton state with a significantly increased probability.

**Fig. 5:**
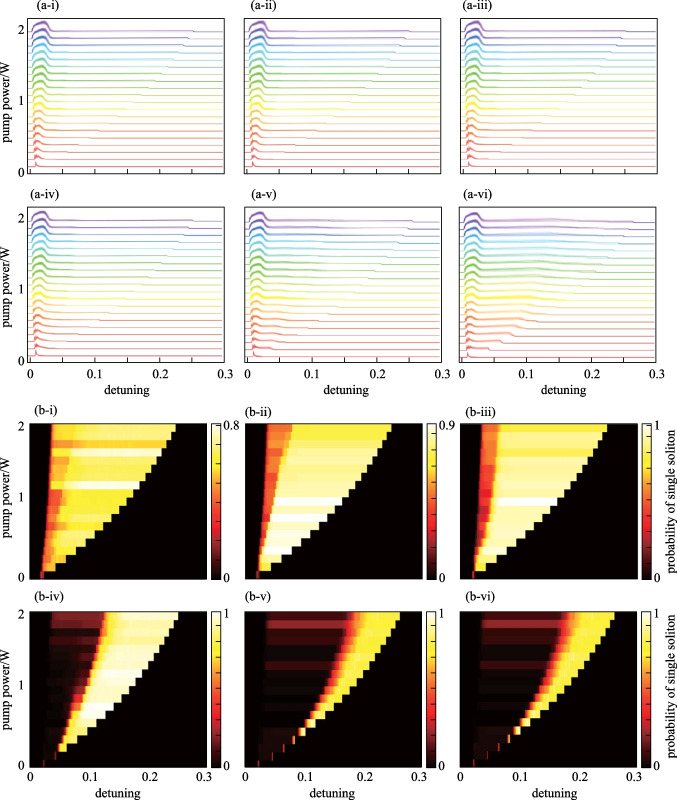
Intracavity power traces and single soliton generation probabilities for depletion at different modes. **a** Superposition of 100 intracavity power traces in each sub-figure. **b** (i–vi) correspond to the cases where mode with $\mu =1, 5, 11, 21, 41, 81$ is depleted. The parameters of the coupled filter are $g=0.15$, ${\sigma }_{f}=\frac{1}{4}\mathrm{FSR}$. **b** Single soliton generation probabilities vs. pump-resonance detuning and pump power, **b** (i − vi) correspond to the cases where mode with $\mu =1, 5, 11, 21, 41, 81$ is depleted. The parameters used are the same as in Fig. 4

Figure 5 gives the impact of spectral separation between the depleted mode and the pump on the probability of single soliton generation. By setting the pump at one of the resonance modes while altering the values of $\mu (1, 5, 11, 21, 41, 81$), 6 cases are considered where the depleted mode is near or far from the pump. Simulation results show that single soliton generation is enhanced for all 6 cases. When the spectral separation between the depleted mode and the pump increases, the energy dissipation gradually diminishes, as the spectrum of single soliton has a standard *Sech*^*2*^ envelope, as shown in Fig. 2c. As a result, the perturbation induced by mode depletion decreases, and enhancement of single soliton generation is less effective. Figure 5a illustrates intracavity power traces vs. different combinations of pump-resonance detuning and pump power. Figure 5b presents single soliton generation probabilities vs. different combinations of pump-resonance detuning and pump power. It is shown that, as the $\mu$ increases, the single soliton existence region gradually becomes smaller. It is worth emphasizing that the single soliton generation is enhanced even under low pump powers.

### Single soliton generation under thermal effect

**Fig. 6:**
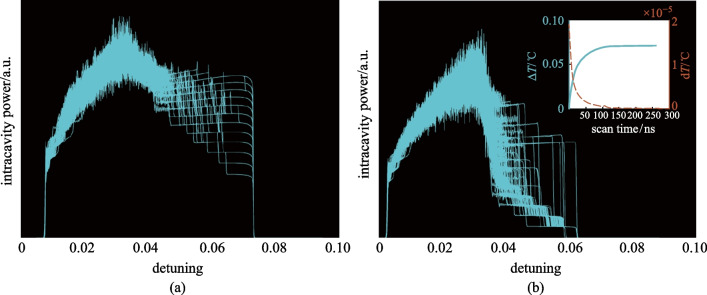
Simulation results of 100 normalized intracavity power traces considering the thermal effect **a** without and **b** with the SMD effect. The inset in **b** gives the temperature variation in the resonator ($\Delta T$, the solid blue curve) and the temperature increment in every round-trip ($\mathrm{d}T$, the green dotted line). Parameters used are the same as Fig. 2. $\delta$ scans linearly starting from 0.005 at a rate of $1.5\times {10}^{-6}$ per round-trip. The thermal parameters of the silicon nitride micro-ring resonator are $K=2.78\times {10}^{-8 }\mathrm{J}/\left(\mathrm{s}\cdot{^\circ \mathrm{C}}\right)$, ${C}_{p}=8.81\times {10}^{-10}\mathrm{ J}/^\circ \mathrm{C}$ and $a\approx 1.43\times {10}^{-5}$

The light-induced thermal effect causes warming of the micro-ring resonator. Due to the thermal-optical effect, the temperature change will lead to the change of the refractive index and thus the change of nonlinear phase-shift. In this subsection, we discuss single soliton generation based on SMD under the influence of the thermal effect. Numerical simulation is conducted using the GLLE with thermal effects [27]:

4 $${t}_{\mathrm{R}}\frac{\partial E\left(t,\tau \right)}{\partial t}=\left[-\alpha -\mathrm{i}\delta -\mathrm{i}L\sum_{k\ge 2}\frac{{\beta }_{k}}{k!}{\left(\mathrm{i}\frac{\partial }{\partial \tau }\right)}^{k}+\mathrm{i}{\gamma L\left|E\right|}^{2}+\mathrm{i}L{{k}_{0}K}_{\mathrm{THO}}\Delta T\right]E-f\left(\tau \right)*E+{\sqrt{{\kappa }_{\mathrm{in}}}E}_{\mathrm{in}},$$

where ${k}_{0}$ is the wave number in vacuum at the central wavelength of the OFC, ${K}_{\mathrm{THO}}$ is the thermal-optical coefficient of the core materials of the ring waveguide and $\Delta T$ is the temperature change of the micro-ring resonator. The phase-shift change of the light field caused by the thermal effect after propagating the unit length can be expressed as $\Delta {\varphi }_{t}={k}_{0}{\Delta n}_{1}={k}_{0}{K}_{\mathrm{THO}}\Delta T\left(t\right)$. Based on the energy conservation principle, the net heat delivered to the micro-ring is the heat that goes in (${q}_{\mathrm{in}}$) minus the heat that goes out (${q}_{\mathrm{out}}$) [28, 29]:

5 $${C}_{p}\frac{\mathrm{d}\Delta T\left(t\right)}{\mathrm{d}t}=\frac{\mathrm{d}{q}_{\mathrm{in}}\left(t\right)}{\mathrm{d}t}-\frac{\mathrm{d}{q}_{\mathrm{out}}\left(t\right)}{\mathrm{d}t}={I}_{h}\frac{1}{{\left(\frac{{\lambda }_{p}-{\lambda }_{0}\left(1+a\Delta T\right)}{\frac{\Delta \lambda }{2}}\right)}^{2}+1}-K\Delta T,$$

where ${C}_{p}$ is the heat capacity of silicon nitride, $K$ is the thermal conductivity of the material between the resonator and environment, ${\lambda }_{p}$ is the wavelength of the pump, ${\lambda }_{0}$ is the cold cavity resonant wavelength of the considered mode, $a$ indicates the thermal coefficient change, ${I}_{h}$ is the actual optical power in the cavity, $\Delta \lambda ={{\lambda }_{p}-\lambda }_{0}$. Figure 6a and b show 100 normalized intracavity power traces obtained without and with SMD, respectively, with the thermal effect taken into consideration. In Fig. 6b, it is noticed that the single soliton generation is enhanced in the vicinity of $\delta =0.06$, compared to that in Fig. 6a. Therefore, the probability of single soliton generation can be greatly improved even if the resonator is affected by the thermal effect.

### Discussion on the soliton existence regime

In the probability map with different combinations of pump-resonance detuning and pump power (it is called *δ*-${E}_{\mathrm{in}}^{2}$ probability map afterward for convenience), it is common to see that the soliton existence region is changed when the selective SMD takes effect. In this section, the stability analysis of the GLLE with a coupled lumped filter is studied by analyzing bifurcation features of the stable solutions of GLLE, and the changes of the soliton existing regime are thus determined [30, 31]. To analyze the dynamics of DKS generation process in our case, a detuning-dependent loss term is introduced to replace the convolution term in the GLLE (Eq. 3), as shown below [31]:

6 $$\frac{\partial E({t}^{\mathrm{^{\prime}}},\tau )}{\partial {t}^{\mathrm{^{\prime}}}}=\left[-\alpha \left(\delta \right)-\mathrm{i}\delta -\mathrm{i}\frac{{\partial }^{2}}{\partial {\tau }^{2}}+\mathrm{i}{\left|E\right|}^{2}\right]E+{E}_{\mathrm{in}}.$$

Note that Eq. (6) is in normalized form. ${t}^{^{\prime}}=t/(2{\tau }_{\mathrm{ph}})$ is dimensionless time where ${\tau }_{\mathrm{ph}}$ is the average photon lifetime which is the reciprocal of resonance linewidth of the micro-ring resonator $\Delta {\omega }_{\mathrm{ring}}$, i.e., ${\tau }_{\mathrm{ph}}=1/\Delta {\omega }_{\mathrm{ring}}$ (see Ref. [30]. for detail). Here $\alpha (\delta )$ is the total energy dissipation, and the energy dissipation caused by filtering is treated as a term proportional to detuning, thus $\alpha \left(\delta \right)={\alpha }_{0}+C\delta$, where ${\alpha }_{0}$ is the intrinsic cavity loss, $C$ indicates the energy dissipation coefficient related to lumped filtering, which can be determined by calculating the energy dissipated as a proportion of the total soliton energy. It is worth noting that the loss term is in a linear form under the first-order perturbation approximation, since the energy of a single comb line only occupies a tiny part of the total soliton energy.

To conduct stability analysis, it is necessary to find all the equilibria of GLLE by setting all the differential terms concerning time to zero, yielding

7 $$\left[-\alpha \left(\delta \right)-\mathrm{i}\delta +\mathrm{i}{\left|E\right|}^{2}\right]E+{E}_{\mathrm{in}}=0.$$

After mathematical deduction, two boundaries of the equilibria denoted by ${E}_{\mathrm{in}-}^{2}(\delta )$ and ${E}_{\mathrm{in}+}^{2}(\delta )$ can be found, which can be expressed in terms of pump-resonance detuning $\delta$

8a $${E}_{\mathrm{in}-}^{2}\left(\delta \right)=\frac{2\delta +\sqrt{{\delta }^{2}-3{\alpha }^{2}\left(\delta \right)}}{3}\left[{\alpha }^{2}\left(\delta \right)+{\left(\frac{\sqrt{{\delta }^{2}-3{\alpha }^{2}\left(\delta \right)}-\delta }{3}\right)}^{2}\right],$$

8b $${E}_{\mathrm{in}+}^{2}\left(\delta \right)=\frac{2\delta -\sqrt{{\delta }^{2}-3{\alpha }^{2}\left(\delta \right)}}{3}\left[{\alpha }^{2}\left(\delta \right)+{\left(\frac{\sqrt{{\delta }^{2}-3{\alpha }^{2}\left(\delta \right)}+\delta }{3}\right)}^{2}\right].$$

**Fig. 7:**
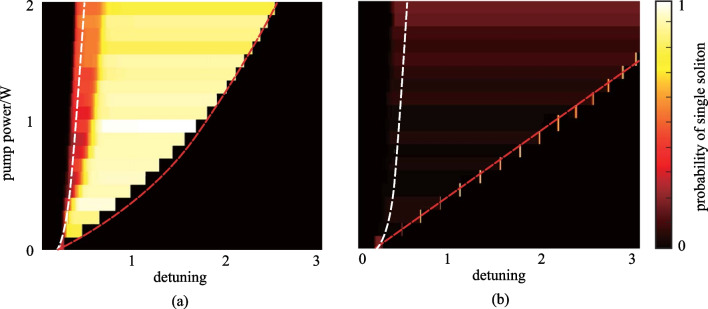
Probability of single soliton generation with different combinations of pump-resonance detuning and pump power **a** with and **b** without filtering. The bifurcation boundaries determined by equilibria of GLLE are also given. White/red dashed lines represent the upper/lower boundary of the bright soliton region determined by Eqs. (8a) and (8b). Parameters used in **a** and **b** are the same as in Fig. 5b-iii and in Fig. 4c. In **a**, the lumped filtering induced energy dissipation coefficient is $C=2.7\times 1{0}^{-3}$

The boundaries described by Eqs. (8a) and (8b) are represented by the white and red dashed lines in Fig. 7, respectively, revealing the bifurcation features of considered GLLE. Figure 7a gives the probability of single soliton generation when the mode with $\mu =11$ is depleted. For comparison, the result without dissipation is presented in Fig. 7b. Figure 7 shows that the simulation results consist of the boundaries determined by Eqs. (8a) and (8b). The results demonstrate that the introduction of the detuning-dependent loss term is valid.

## Conclusions

In conclusion, we theoretically demonstrated a method of realizing single soliton generation with an extremely high probability of up to 98% even at low pump powers, by selectively dissipating a spectral mode through coupling a filter to the micro-ring resonator. The feasibility of using an on-chip spectral filter for spectral mode depletion in a micro-ring resonator was studied. Then, the dynamics of DKS generation with lumped filtering were simulated using the GLLE. We showed that single mode depletion introduced by the coupled filter could boost single soliton generation probability in the anomalous GVD regime significantly. We also showed the robustness of the method by verifying that probability of single soliton generation can be improved, even evolving the thermal effect. Meanwhile, the bifurcation analysis of the normalized GLLE was carried out to define the soliton existence region. With mode depletion, we showed that the soliton existence region is changed. Moreover, filtering strategy can offer an environmentally friendly solution as it can be implemented using all-passive devices. For comparison, the Zeno effect-induced mode coupling is an active operation requiring extra pump light [26]. Moreover, our scheme only requires linear pump tuning, and does not need dispersion engineering. It is also suitable for various material platforms. Our findings may lead to further explorations in the area of on-chip OFC, and the proposed scheme can be a good candidate for a wide range of applications such as optical soliton communications, spectroscopy, and beyond.

## Data Availability

The data that support the findings of this study are available from the corresponding author, upon reasonable request.
